# Effects of Dietary Organic and Inorganic Sulfur on Laying Performance, Egg Quality, Ileal Morphology, and Antioxidant Capacity in Laying Hens

**DOI:** 10.3390/ani12010087

**Published:** 2021-12-31

**Authors:** Yoo-Bhin Kim, Sang-Hyeok Lee, Da-Hye Kim, Hyun-Gwan Lee, Yongjun Choi, Sung-Dae Lee, Kyung-Woo Lee

**Affiliations:** 1 Department of Animal Science and Technology, Konkuk University, Gwangjin-gu, Seoul 05029, Korea; ybin51@naver.com (Y.-B.K.); the_shinism@naver.com (S.-H.L.); kdh142536@naver.com (D.-H.K.); leehyun3177@naver.com (H.-G.L.); cyj2114@gmail.com (Y.C.); 2 National Institute of Animal Science, Rural Development of Administration (NIAS-RDA), Wanju 55365, Korea; leesd@korea.kr

**Keywords:** methyl sulfonyl methane, sodium sulfate, laying hen, antioxidant capacity

## Abstract

**Simple Summary:**

Oxidative stress caused by environmental and nutritional factors could be detrimental to poultry production. Dietary natural antioxidants could therefore be beneficial in limiting the deleterious effects of oxidative stress in chickens. Methyl sulfonyl methane is a non-toxic natural organosulfur compound with the chemical formula (CH_3_)_2_SO_2_ and is known as methyl sulfone or dimethyl sulfone. Inorganic sulfate (e.g., sodium sulfate) is involved in the metabolism of many tissues and systems, as well as in important detoxication mechanisms. Dietary sulfur in either organic or inorganic forms exhibits beneficial antioxidant properties in various animals in vivo and in vitro. Therefore, our studies have been conducted to evaluate the role of organic and inorganic sulfur in laying hens.

**Abstract:**

The present study was conducted to investigate the comparative effects of organic and inorganic forms of sulfur, methyl sulfonyl methane (MSM) and sodium sulfate (SS), on laying performance, egg quality, ileal morphology, ileal volatile fatty acids, and antioxidant and stress markers in various biological samples in aged laying hens. A total of 144, 73-week-old Lohman Brown-Lite laying hens were randomly assigned to one of three experimental diets: basal diet (CONT), CONT + 0.2% MSM (MSM), and CONT + 0.3% SS (SS). The trial lasted for 12 weeks. MSM and SS diets contained 0.07% of sulfur, either organic or inorganic. Dietary MSM did not affect egg production or feed conversion ratio at 12 weeks compared with the CONT group. Dietary sulfur did not affect egg quality except for the Haugh unit at 4 weeks, which was lowered (*p* < 0.05) in the SS group. Compared with the CONT group, a higher (*p* < 0.05) villus height to crypt depth ratio was observed in the SS group. Dietary sulfur did not affect the percentages of short-chain fatty acids in the ileum. Total antioxidant capacity of the liver increased (*p* < 0.05) in laying hens fed MSM- and SS-added diets compared with the CONT group. The MSM and SS groups were found to have lowered (*p* < 0.05) malondialdehyde (MDA) concentration in serum samples compared with CONT. Finally, dietary MSM had the lowest (*p* < 0.05) MDA concentrations in yolk samples. Taken together, our study showed that dietary organic and inorganic sulfur have positive effects on ileal morphology and antioxidant capacity in laying hens. However, SS-mediated inhibition in laying performance needs to be clarified.

## 1. Introduction

Oxidative stress defines a disturbed balance between production of free radicals and their elimination by the antioxidant defense systems [1]. It has been well acknowledged that oxidative stress caused by environmental and nutritional factors could cause production loss in poultry [2]. Thus, dietary supplementation of natural or synthetic antioxidants in the diets of chickens has been a nutritional strategy to reduce oxidative damage [3]. There are demands for using natural products that can prevent lipid oxidation in fat-enriched animal foods due to consumer preferences for natural substances and toxicological concerns about synthetic antioxidants [4]. The natural antioxidant can fully replace the synthetic antioxidant function and be used as a valuable source for reducing oxidative stress [5,6]. Thus, dietary natural antioxidants could be beneficial in limiting the deleterious effects of oxidative stress in chickens [7,8].

Methyl sulfonyl methane (MSM), also known as DMSO_2_, or organic sulfur, is an antioxidant agent [9] and is naturally occurring in plants and animals. Dietary MSM has been used to improve antioxidant capacity and to reduce inflammation, joint/muscle pain, and oxidative stress in humans [10,11]. It has been reported that MSM, as a sulfur supplement, is nontoxic upon consumption, and is a safer source of sulfur compared with sulfur-containing amino acids (methionine and cyst(e)in), which are toxic at a high intake [12]. In addition to its antioxidant capacity, MSM has been reported to possess various biological activities, being associated with antimicrobials and immune modulations in mice [13], ducks [14], and laying hens [15]. 

Sodium sulfate anhydrous (SS), inorganic sulfur, is used as a viscosity-increasing agent in cosmetic formulations [16]. As sulfate is involved in various metabolic and detoxication processes, it seems reasonable to add an adequate level of sulfur in the diet [17]. Reid and Weber [18] reported that the requirement of sulfur-containing amino acids in chickens could be partially met by dietary supplementation of inorganic sulfate, indicating the possibility of replacing the pools of methionine or cysteine. Wong et al. [12] suggested that inorganic sulfate could also be used for sulfation of acetaminophen to reduce toxicity and for sulfation of mucin secreted by the intestine, which is incorporated into various tissues. In addition, dietary inorganic sulfur has been known to possess various biological activities, including antioxidant, anti-inflammatory, and antibiotic activities in pigs [19] and broilers [20]. 

It is thus clear that dietary sulfur in either organic or inorganic forms exhibits beneficial antioxidant properties in various animals in vivo and in vitro [21,22]. However, the antioxidant role of sulfur [23] may not be attributable to its structure due to the lack of direct quenching oxidant activity and of functional groups (e.g., hydroxy groups) [24]. In addition, no studies have been conducted to evaluate the role of organic and inorganic sulfur in laying hens. Therefore, we attempted to investigate the effects of MSM and SS, as sources of organic and inorganic sulfur, on laying performance, egg quality, and antioxidant capacity in laying hens. As sulfur exhibits antimicrobial properties [25], gut health and stress indicators, including ileal morphology and ileal short-chain fatty acids (SCFAs) and corticosterone in eggs, were also analyzed. It was anticipated that the information obtained would be useful in assessing the value of adding sulfur to the feed of laying hens and could lead to the construction of hypotheses to be tested in further studies.

## 2. Materials and Methods

### 2.1. Test Materials

Methyl sulfonyl methane (Sigma-Aldrich, St. Louis, MO, USA) is in the form of a white crystalline powder and contains 34.1% sulfur on a weight basis. MSM contents in the basal and experimental diets were measured using gas chromatography as described by Park and Lee [26]. 

Sodium sulfate (Samchun Chemicals, Pyeongtaek, Korea) is an inorganic compound with the formula Na_2_SO_4_ and contains 22.6% sulfur on a weight basis. Both MSM and SS are white solids that are highly soluble in water.

### 2.2. Birds and Experimental Design

A total of 144, 73-week-old laying hens (Lohmann Brown-Lite) were randomly assigned to one of three dietary groups with eight replicates per group. Two hens were raised in a cage (45 cm × 45 cm × 45 cm) in a windowless, fan-ventilated house, and the adjacent three cages were considered as replicates (*n* = 6 birds/replicate). During an experimental period of 12 weeks, laying hens were fed corn and soybean meal-based diets supplemented without (CONT) or with equal amounts of sulfur (0.7 g S/kg of diet) from MSM (2.0 g/kg of diet) or SS (3.0 g/kg of diet). We decided to choose 0.7 g S/kg of diet based on an earlier study showing that dietary MSM at 0.2% (0.7g S/kg of diet) improved egg quality and enhanced cell-mediated immune response in laying hens [15]. Sulfur contents in the basal and experimental diets were measured with an elemental analyzer (EA 1110 CHN; CE instruments, Rodano, MI, Italy). The analyzed total sulfur contents of the CONT, MSM, and SS diets were 0.15, 0.31, and 0.30%, respectively. The aged laying hens in this study were chosen as oxidative stress increases with the age of laying hens [27]. 

As SS contains 32.4% sodium, when added at 3.0 g/kg, the SS diet provided an extra 0.97 g Na/kg of diet. Thus, sodium bicarbonate was added in the CONT and MSM diets at 3.5 g per kg of diet to provide amounts of sodium equal to the SS diet. MSM, SS, and sodium bicarbonate were first pre-mixed with a carrier before mixing them with the basal diet to formulate the experimental diets. The ingredients and composition of the basal diet are shown in Table 1. All experimental diets were formulated to meet or exceed the nutrient requirements of aged brown egg-laying hens as recommended by the Korean Feeding Standards for Poultry [28]. The MSM contents were analyzed to contain 0.03%, 0.17%, and 0.03% in the CONT, MSM, and SS diets, respectively. 

Feed and water were supplied to allow ad libitum consumption during the experimental period. A lighting program of 15 h of light and 9 h of dark was applied for the entire experimental period. The temperature and relative humidity in the experimental room were maintained at 21 ± 2 °C and 60%.

### 2.3. Laying Performance and Egg Quality

Feed consumption per replicate was recorded monthly and used to calculate daily feed intake per bird. Egg production and egg weight were daily recorded and used to calculate the egg mass. The percentage of dirty and broken eggs was calculated as (total number of dirty and broken eggs per replicate/total number of eggs per replicate) × 100. The feed conversion ratio was calculated as feed intake/egg mass per replicate. 

On the last three consecutive days at 4, 8, and 12 weeks, six intact eggs per replicate were collected for egg quality assessment. Eggshell color was estimated with a shell color reflectometer (TSS QCR, Technical Services and Supplies, York, UK). Haugh unit, eggshell strength, eggshell thickness (without shell membrane), and yolk color score were assessed with a digital egg tester (DET−6000, Nabel, Kyoto, Japan). Yolk color was automatically graded on a scale of 1–16, with 1 being a very pale yellow and 16 being a dark orange. The separated yolks were weighed after clearing adherent albumin residues with filter paper [29]. Eggshells were cleaned to remove the adherent albumen, dried at room temperature for 3 days, and weighed. Albumen weight was then calculated by subtracting yolk and dry eggshell weights from the initial egg weight.

### 2.4. Corticosterone and Malondialdehyde in Egg Yolk

Three eggs per replicate were collected for the determination of corticosterone in egg yolks at 4, 8, and 12 weeks. The eggs were broken, yolk was separated from albumen, and separated yolks were placed in plastic bags. The pooled yolk per replicate was homogenized and used to measure corticosterone (Enzo Life Sciences, Inc., ADI-901-097, Farmingdale, NY, USA). Pooled yolks were then centrifuged at 1200× *g* for 5 min at 4 °C. The supernatants were analyzed for malondialdehyde (MDA; Cell Biolabs, Inc., San Diego, CA, USA) per the recommendation by the manufacturers.

### 2.5. Ileal Morphology

At 12 weeks, one hen per replicate was euthanized with an overdose of carbon dioxide. Immediately after euthanasia, the small intestine was excised, and a segment of mid-ileum was sampled. Approximately, a 1 cm-long mid-segment of the ileum was fixed in 10% neutral buffered formalin for 48 h, dehydrated, and embedded in a paraffin block. Histological sections (5 µm thick) were stained with hematoxylin and eosin per standard histological technique. The mucosa was examined by a light microscope (Olympus BX43, Tokyo, Japan) and photographed using a digital camera (eXcope T500, DIXI Science, Daejeon, Korea). Ten intact well-oriented villi and crypts were counted for villus height and crypt depth. Villus height was measured from the villus tip to the villus bottom and crypt depth was defined from villus bottom to the crypt. The ratio of villus height and crypt depth was then calculated. 

### 2.6. Short-Chain Fatty Acids Analysis

At the end of the experiment (i.e., 12 weeks), approximately 1 g of ileal digesta was sampled from one bird per replicate and thoroughly homogenized in 4 mL of cold distilled water using a vortex mixer. The homogenate was then added to 0.05 mL of saturated HgCl_2_, 1 mL of 25% H_3_PO_4_, and 0.2 mL of 2% pivalic acid and centrifuged at 1000× *g* at 4 °C for 20 min. To measure the concentrations of SCFAs by gas chromatography (6890 Series GC System; HP, Palo Alto, CA, USA) as described by Kim et al, 1 mL of supernatant was used [30]. 

### 2.7. Antioxidant Markers in Liver and Serum Samples

At 12 weeks, livers were sampled and stored on ice until further preparation on the day of the sampling. Approximately 1 g of liver was mixed in 9 mL of cold 1X PBS and homogenized (Digital Ultra-Turrax T25, IKA, Staufen, Germany). The homogenate was then centrifuged at 10,000× *g* for 10 min, and the aliquot of the supernatant was stored at –20 °C until analysis. The diluted aliquot was used for the determination of glutathione peroxidase (GPX; EnzyChrom GPx, BioAssay Systems, Hayward, CA, USA), total antioxidant capacity (TAC; QuantiChrom Antioxidant, BioAssay Systems, Hayward, CA, USA), catalase (CAT; Cell Biolabs, Inc., San Diego, CA, USA), and MDA (Cell Biolabs, Inc., San Diego, CA, USA) per the instructions described by the manufacturers. The results were normalized against total protein concentration in each sample. Total protein concentration in liver was quantified as described by Bradford [31] using bovine serum albumin. 

Approximately 3 mL of blood per hen (one hen per replicate) was drawn from the wing vein into the clot activator tube at 4, 8, and 12 weeks. Serum samples were obtained by gentle centrifugation (200× *g*) for 15 min and stored at –20 °C before analysis. Serum samples were used to measure various biomarkers of oxidative stress, including levels of GPX, superoxide dismutase (SOD), TAC, CAT, MDA, and 8-hydroxydeoxyguanosine (8-OHdG). SOD was analyzed using an SOD determination assay kit-WST (Sigma, St. Louis, MO, USA) and expressed as SOD activity (inhibition rate, %). As an indicator of oxidative DNA damage, 8-OHdG was determined using an 8-OHdG DNA Damage ELISA Kit (Cell Biolabs, Inc., San Diego, CA, USA) and was presented in ng/mL. All assays were conducted per the recommendations specified by the manufacturers.

### 2.8. Statistical Analysis

All data were analyzed by one-way ANOVA using PROC GLM (v9.4; SAS Institute Inc., Cary, NC, USA). Treatment means were separated using Duncan’s multiple range test [32]. The significance level was pre-set at *p* < 0.05.

## 3. Results

### 3.1. Laying Performance and Egg Quality

The production performance of laying hens fed diets with different sulfur sources is presented in Table 2. At weeks 4 and 8, feed intake was lowest (*p* < 0.05) in the SS group compared with the CONT and MSM groups. SS vs. MSM significantly lowered (*p* < 0.05) feed intake at 4 weeks. The SS-mediated depression in feed intake was not noted (*p* > 0.05) by the time of 12 weeks. Egg production was lowest (*p* < 0.05) in the SS group compared with the CONT and MSM groups at 8 and 12 weeks. Feed conversion ratio was significantly elevated (*p* < 0.05) in the SS-fed laying hens compared with the CONT and MSM groups at 8 and 12 weeks.

Neither MSM nor SS significantly affected the percentages of dirty and cracked eggs at any ages (*p* > 0.05; Table 3). SS-fed laying hens laid lighter eggs by on average 4.0% at 4 weeks (*p* < 0.05) and 3.1% at 8 weeks (*p* > 0.05) compared with the CONT. No difference in egg weight was noted between dietary treatments at 12 weeks. At 8 and 12 weeks, egg mass was lowest (*p* < 0.05) in the SS group compared with the CONT and MSM groups (Table 3).

To address the question of whether different sulfur sources would affect internal egg qualities, the compositions and qualities of eggs were monitored at 4-week intervals. None of the dietary sulfur treatments affected egg compositions and qualities except for the Haugh unit at 4 weeks (Table 4). The Haugh unit was lower (*p* < 0.05) in the SS group compared with the CONT and MSM groups at 4 weeks. 

### 3.2. Corticosterone and MDA in Yolk Samples

No difference in yolk corticosterone between dietary treatments was noted at 4, 8, and 12 weeks (Table 5). Dietary MSM did not affect (*p* > 0.05) the MDA contents of egg yolks at 4 and 8 weeks compared with the CONT. However, at 12 weeks, MSM-fed, not SS-fed, laying hens had the lowest MDA contents (*p* < 0.05) in egg yolks compared with the CONT group.

### 3.3. Ileal Morphology and Ileal SCFA Concentration

Dietary sulfur treatments did not affect ileal villus height and crypt depth (Table 6). However, villus height:crypt depth ratio was elevated by on average 1.3- and 1.7-fold in the MSM and SS groups compared with the CONT. None of the dietary sulfur treatments affected the relative percentages of SCFAs in ileal digesta at 12 weeks (Table 7).

### 3.4. Markers for Oxidative Stress in Liver Samples

None of the dietary sulfur sources affected (*p* > 0.05) GPX activity, CAT, and MDA levels in liver (Table 8). It was observed that GPX activity ranged from 51.8 to 59.7 U per mg of protein, CAT from 65.2 to 70.8 U per mg of protein, and MDA from 1.98 to 2.35 nmol per mg of protein. The TAC levels in liver were significantly elevated in both MSM and SS groups compared with the CONT group at 12 weeks (*p* < 0.05). 

### 3.5. Markers for Oxidative Stress in Serum Samples

Dietary sulfur sources did not affect GPX activity, TAC, CAT, or 8-OHdG in serum samples at all ages (Table 9). Although statistically non-significant, MSM-fed chickens had the highest SOD activity (*p* > 0.05) by on average 28.6% and 21.2% at 4 and 12 weeks compared with the CONT group. Laying hens fed diets containing SS vs. MSM showed (*p* > 0.05) similar, higher, or slightly lower SOD activities at 4, 8, and 12 weeks. At 12 weeks, both MSM and SS groups had lower (*p* < 0.05) MDA concentrations in serum samples compared with the CONT group.

## 4. Discussion

Our study showed that laying hens fed diets containing inorganic vs. organic sulfur performed less well, as manifested by the deterioration in feed intake, egg production, feed conversion ratio, egg weight, and egg mass. This finding was unexpected in the light of earlier studies in which impaired laying performance due to SS was only noted at the higher added concentrations of 10,000 ppm in the diet [33] or 16,000 ppm in drinking water [34]. It was also reported that dietary sulfate at 1.0% did not affect egg production and feed intake in laying hens [35]. Furthermore, SS is considered a less toxic sulfur source compared with magnesium sulfate in laying hens [34]. Finally, Ross et al. [36] showed that the addition of SS in a broiler diet at the level of 0.3% increased body weight gain compared with the no-added diet-fed control. In this sense, the negative effect of dietary SS that emerged from our study is not to be explained in terms of the SS level having been toxic for laying hens. In addition, SS is used in cosmetic formulations as a viscosity-increasing agent and listed as generally recognized as safe (GRAS) by the Food and Drug Administration [37].

Alternatively, the SS-depressed laying performance might be related to differences in sodium sources, although all treatment groups received equal amounts (1.9 g Na/kg of diet). The control group received sodium from sodium bicarbonate plus NaCl while the SS group received it from NaCl plus SS. Indeed, Ahmad et al. [38] reported that dietary SS vs. sodium bicarbonate inhibited water intake in heat-stressed broiler chickens. Thus, it needs to be addressed whether the SS-mediated inhibition of feed intake could be linked to altered water intake or dynamics. In contrast to SS, dietary MSM did not affect laying performance albeit that both SS and MSM were given equal amounts of sulfur. The lack of effect of dietary MSM on performance was reported with laying hens [15] and broiler chickens [11,39]. 

None of the dietary sulfur sources affected egg quality except for the Haugh unit. Of note, the Haugh unit, an indicator of internal egg quality, was low in SS-fed groups at 4 and 8 weeks. In contrast to our finding, Adams et al. [34] found that SS in drinking water up to 16,000 ppm did not affect the Haugh unit and eggshell thickness in laying hens. A clear explanation is not readily available as to the SS-mediated decrease in the Haugh unit. As the experimental diets contained equal amounts of sulfur, diet-origin sulfur per se in eggs might not be the factor affecting the Haugh unit. Thus, whether an SS-induced decrease in the Haugh unit is related to its impact on albumen components (i.e., ovomucin or lysozyme contents in thick albumen) needs to be addressed. 

Corticosterone, a well-known stress hormone in poultry, plays an important role in suppressing immune responses and animal performance [40]. Corticosterone is accumulated in eggs in a chronic manner before ovulation [41]. The concentration of corticosterone detected in this study was within the physiological range, being from 12.82 to 1033 pg in egg yolks [42], indicating a negligible effect of sulfur on stress response in laying hens. 

The structure and functionality of the intestinal microbiota are crucial for the health of poultry. Due to the antimicrobial activities of MSM [43] and SS [44], it is expected that their supplementation in the diets of laying hens could balance gut microbiota and improve gut health, which prompted us to measure ileal morphology and ileal SCFAs. Both MSM and SS did not affect ileal SCFAs, but increased villus height and crypt depth ratio, the indicator of gut function and health [45,46]. We found that SS vs. MSM was more effective in increasing villus height and crypt depth ratio. Our study is in line with earlier findings [20] showing that duodenal villus height and crypt depth ratio was increased in broilers fed diets to which 2 or 3 g S/kg of feed were added. Scott et al. [44] indicated that dietary SS was an effective antimicrobial intervention to reduce *Salmonella* contamination. However, the contradictory finding that SS-induced improvement in gut morphology led to a negative effect on laying performance precludes a conclusion of the positive effect of dietary SS in laying hens. In future, the sulfur-mediated effect on intestinal physiology and health warrants further studies addressing the role of organic vs. inorganic sulfur on gut microbiomes and gut barrier integrity. 

Due to the role of sulfur-containing substances as regulators in oxidative stress [23,47], it is expected that both MSM and SS could possess antioxidative properties. Thus, we attempted to measure enzymatic and non-enzymatic antioxidative systems in various biological samples, including eggs, liver, and serum samples. We found that dietary MSM vs. SS was more effective in reducing yolk MDA concentrations. Both MSM and SS raised TAC and SOD activity but lowered MDA in serum samples compared with the CONT. Tentatively, our findings indicate that both MSM and SS have potential as antioxidant feed additives in laying hens, although the analyzed antioxidant and oxidative stress parameters were not closely associated. 

MDA is a major oxidation product of peroxidized polyunsaturated fatty acids and an important indicator of lipid peroxidation [48]. Dietary MSM has been known to show a wide spectrum of antioxidant activity in humans [49], pigs [50], Pekin ducks [14], and broiler chickens [51,52]. In this sense, our observation that dietary SS decreased MDA concentrations in serum samples might suggest the quenching activity of diet-origin sulfur per se in oxidative stress. However, it seems that there would be other factors in addition to sulfur itself affecting the antioxidative/oxidative balance as yolk MDA concentration was only affected by MSM but not SS. TAC is used to assess the antioxidant status of the body, reflecting all the antioxidant substances present in biological samples [53]. We noted that the concentrations of TAC in liver samples, but not in serum samples, were elevated in laying hens fed diets containing dietary sulfur (both MSM and SS). It has been reported that dietary MSM increased plasma/serum concentrations of TAC in human subjects [49] and Pekin ducklings [14]. However, it should be pointed out that a sulfur-induced increase in TAC concentration was not associated with a concomitant decrease in MDA concentration in liver samples. It is not surprising to see the inconsistent results for dietary antioxidants, including MSM, with oxidant–antioxidant defense biomarkers [52]. 

SOD is a powerful antioxidant in the cell and an important endogenous antioxidant enzyme acting to suppress or prevent the formation of free radicals [54]. Earlier studies also showed that dietary MSM at the level of 0.3% increased SOD activity in serum samples of ducks [14,55]. However, dietary MSM did not affect SOD activity although it increased the activity by 21.2% at 12 weeks compared with the CONT group. In contrast to previous studies reporting the beneficial effect of dietary MSM on GPX in broilers, ducklings, and horses [14,52,56], we did not observe an effect of dietary MSM or SS on GPX activity in serum and liver. However, as far as we know, this is the first report to compare the effect of dietary sulfur originated from either organic or inorganic sources on antioxidant capacities in laying hens. 

## 5. Conclusions

In conclusion, dietary SS impaired laying performance (i.e., there was a reduction in feed intake and egg production), but improved ileal morphology (i.e., villus height:crypt depth ratio). Both SS and MSM exhibited antioxidative activity. Collectively, our study suggests that dietary sulfur can be used as a potential feed additive to mitigate oxidative stress and to improve the gut health of laying hens, which seems to be beneficial for poultry. Future studies are required to investigate how SS might have inhibited feed (or water) intake and how dietary sulfur might affect gut microbiota in laying hens.

## Figures and Tables

**Table 1:** Ingredient and nutrient composition of the basal diet.

Ingredients	g per 100 g of Diet
Corn	41.00
Soybean meal, 45% crude protein	10.41
Wheat	12.80
Animal fat	1.02
Rice bran	2.00
Corn steep liquor	1.00
Rapeseed meal	3.00
Dried distillers grains with solubles	12.83
Molasses	2.00
Liquid choline, 50%	0.06
Limestone	10.51
Monodicalcium phosphate	1.02
NaCl	0.24
Variable	1.59
Methionine, 100%	0.07
Lysine, 54%	0.10
Tryptophan, 10%	0.10
Mineral mix ^1^	0.14
Vitamin mix ^2^	0.12
Total	100.00
Nutrient composition, g/100 g	
Nitrogen-corrected apparent metabolizable energy, kcal/kg	2600
Dry matter ^3^	88.20
Crude protein ^3^	14.49
Crude fat ^3^	4.01
Crude ash ^3^	14.84
Calcium ^4^	4.10
Sulfur ^3^	0.15
Available phosphorus ^4^	0.28
Total lysine ^4^	0.65
Total methionine ^4^	0.32
Total methionine + cysteine ^4^	0.60
Methyl sulfonyl methane ^3^	0.03

^1^ Mineral mixture provided the following nutrients per kg of diet: Fe (FeSO_4_.H_2_O), 50 mg; Cu (CuSO_4_.H_2_O), 24 mg; Zn (ZnSO_4_.H_2_O), 90 mg; Mn (MnSO_4_.H_2_O), 96 mg; I (Ca(IO_3_)_2_), 1.2 mg. ^2^ Vitamin mixture provided the following nutrients per kg of diet: vitamin A, 15,400 IU; vitamin D3, 3080 IU; vitamin E, 14 mg; vitamin K3, 1.4 mg; vitamin B1, 1.12 mg; vitamin B2, 2.8 mg; vitamin B6, 3.92 mg; vitamin B12, 0.014 mg. ^3^ Analyzed value. ^4^ Calculated value.

**Table 2:** Effects of dietary sulfur on production performance in laying hens (*n* = 8).

Item	CONT ^1^	MSM	SS	SEM ^2^	*p*-Value
Feed intake, g/bird					
4 weeks	107.6 ^a^	101.4 ^b^	98.2 ^c^	0.91	<0.001
8 weeks	102.0 ^a^	103.9 ^a^	95.7 ^b^	1.75	0.011
12 weeks	107.7	108.2	109.7	2.09	0.839
Egg production, %					
4 weeks	81.9	82.4	78.7	1.89	0.405
8 weeks	85.3 ^a^	83.4 ^a^	75.9 ^b^	1.44	0.001
12 weeks	81.5 ^a^	83.2 ^a^	72.4 ^b^	1.59	0.002
Feed conversion ratio, kg/kg					
4 weeks	2.08	1.98	2.06	0.04	0.332
8 weeks	1.87 ^b^	1.94 ^a,b^	2.03 ^a^	0.04	0.022
12 weeks	2.08 ^b^	2.01 ^b^	2.39 ^a^	0.05	0.001

^a, b, c^ Means value without a common superscript within the same row differ (*p* < 0.05). ^1^ CONT, basal diet; MSM, basal diet + methyl sulfonyl methane; SS, basal diet + sodium sulfate. ^2^ SEM, standard error of the mean.

**Table 3:** Effects of dietary sulfur on dirty and cracked eggs, egg weight, and egg mass in laying hens (*n* = 8).

Item	CONT ^1^	MSM	SS	SEM ^2^	*p*-Value
Dirty and cracked egg, %					
4 weeks	2.17	2.16	2.68	0.67	0.875
8 weeks	2.74	1.69	2.50	0.90	0.697
12 weeks	2.14	1.96	3.40	0.85	0.559
Egg weight, g/egg					
4 weeks	63.42 ^a^	62.21 ^a,b^	60.90 ^b^	0.48	0.014
8 weeks	64.23	64.17	62.22	0.72	0.114
12 weeks	63.82	64.70	63.41	0.73	0.575
Egg mass, g/day					
4 weeks	51.8	51.2	47.9	1.08	0.062
8 weeks	54.8 ^a^	53.5 ^a^	47.2 ^b^	1.01	<0.001
12 weeks	51.9 ^a^	53.9 ^a^	45.9 ^b^	0.96	0.001

^a, b^ Means value without a common superscript within the same row differ (*p* < 0.05). ^1^ CONT, basal diet; MSM, basal diet + methyl sulfonyl methane; SS, basal diet + sodium sulfate. ^2^ SEM, standard error of the mean.

**Table 4:** Effects of dietary sulfur on egg composition and egg quality in laying hens (*n* = 8).

Item	CONT ^1^	MSM	SS	SEM ^2^	*p*-Value
4 weeks					
Relative yolk weight, %	26.14	25.60	25.62	0.28	0.339
Relative eggshell weight, %	9.91	10.07	10.15	0.11	0.338
Relative albumen weight, %	63.94	64.39	64.21	0.29	0.574
Yolk color	6.00	6.02	6.03	0.06	0.952
Haugh unit	76.3 ^a^	78.7 ^a^	72.8 ^b^	0.97	0.003
Eggshell strength, kg/cm^2^	4.90	4.99	4.89	0.20	0.919
Eggshell thickness, mm	0.43	0.43	0.43	0.00	0.560
Eggshell color, unit	28.36	27.89	27.23	0.76	0.651
8 weeks					
Relative yolk weight, %	26.67	26.07	26.32	0.26	0.311
Relative eggshell weight, %	9.94	9.96	10.15	0.12	0.515
Relative albumen weight, %	63.38	63.95	63.52	0.29	0.417
Yolk color	6.75	6.60	6.66	0.09	0.551
Haugh unit	76.7	76.1	73.7	0.91	0.107
Eggshell strength, kg/cm^2^	4.82	4.74	4.47	0.15	0.308
Eggshell thickness, mm	0.41	0.42	0.42	0.00	0.452
Eggshell color, unit	27.37	28.00	27.04	0.51	0.501
12 weeks					
Relative yolk weight, %	25.88	25.43	25.72	0.29	0.572
Relative eggshell weight, %	9.96	9.90	10.15	0.09	0.265
Relative albumen weight, %	64.14	64.67	63.88	0.32	0.311
Yolk color	6.80	6.70	6.95	0.10	0.309
Haugh unit	75.4	77.5	74.9	1.00	0.238
Eggshell strength, kg/cm^2^	4.56	4.62	4.66	0.11	0.974
Eggshell thickness, mm	0.42	0.40	0.41	0.01	0.307
Eggshell color, unit	28.91	28.20	26.68	0.80	0.228

^a, b^ Means value without a common superscript within the same row differ (*p* < 0.05). ^1^ CONT, basal diet; MSM, basal diet + methyl sulfonyl methane; SS, basal diet + sodium sulfate. ^2^ SEM, standard error of the mean.

**Table 5:** Effects of dietary sulfur on corticosterone and malondialdehyde contents of egg yolks in laying hens (*n* = 8).

Item ^1^	CONT ^2^	MSM	SS	SEM ^3^	*p*-Value
Corticosterone, pg/g					
4 weeks	325.6	403.1	429.3	38.81	0.240
8 weeks	195.1	236.7	249.2	15.67	0.088
12 weeks	325.7	325.7	330.8	30.84	0.993
MDA, nmol/g					
4 weeks	33.11	30.83	33.68	0.83	0.064
8 weeks	30.00	28.21	30.89	0.83	0.115
12 weeks	29.08 ^a^	21.77 ^b^	27.16 ^a^	0.87	<0.001

^a, b^ Means value without a common superscript within the same row differ (*p* < 0.05). ^1^ MDA, malondialdehyde. ^2^ CONT, basal diet; MSM, basal diet + methyl sulfonyl methane; SS, basal diet + sodium sulfate. ^3^ SEM, standard error of the mean.

**Table 6:** Effects of dietary sulfur on ileal morphology in laying hens (*n* = 8).

Item ^1^	CONT ^2^	MSM	SS	SEM ^3^	*p*-Value
Villus height, µm	807.4	905.7	1059.0	96.00	0.285
Crypt depth, µm	146.0	128.6	116.4	8.69	0.136
VH:CD ratio	5.44 ^b^	7.15 ^a,b^	9.18 ^a^	0.73	0.030

^a, b^ Means value without a common superscript within the same row differ (*P* < 0.05). ^1^ VH:CD ratio, villus height to crypt depth ratio. ^2^ CONT, basal diet; MSM, basal diet + methyl sulfonyl methane; SS, basal diet + sodium sulfate. ^3^ SEM, standard error of the mean.

**Table 7:** Effects of dietary sulfur on the percentages (%) of ileal short-chain fatty acids in laying hens (*n* = 8).

Item	CONT ^1^	MSM	SS	SEM ^2^	*p*-Value
Acetate	55.86	62.01	59.59	3.22	0.421
Propionate	6.61	5.70	7.17	0.61	0.290
Isobutyrate	5.02	5.11	6.11	1.06	0.754
Butyrate	5.97	5.05	5.55	0.75	0.695
Isovalerate	4.01	4.22	4.23	0.45	0.931
Valerate	4.80	3.83	4.84	0.62	0.455
Lactate	17.74	14.09	12.51	2.71	0.422

^1^ CONT, basal diet; MSM, basal diet + methyl sulfonyl methane; SS, basal diet + sodium sulfate. ^2^ SEM, standard error of the mean.

**Table 8:** Effects of dietary sulfur on oxidative stress markers of liver in laying hens (*n* = 8).

Item ^1^	CONT ^2^	MSM	SS	SEM ^3^	*p*-Value
GPX activity, U/mg protein	51.83	54.38	59.71	2.59	0.224
TAC, nmol/mg protein	52.29 ^b^	63.05 ^a^	65.40 ^a^	1.85	<0.001
CAT, U/mg protein	70.82	65.22	69.93	13.58	0.965
MDA, nmol/mg protein	2.05	1.98	2.35	0.19	0.453

^a, b^ Means value without a common superscript within the same row differ (*p* < 0.05). ^1^ GPX, glutathione peroxidase; TAC, total antioxidant capacity; CAT, catalase; MDA, malondialdehyde. ^2^ CONT, basal diet; MSM, basal diet + methyl sulfonyl methane; SS, basal diet + sodium sulfate. ^3^ SEM, standard error of the mean.

**Table 9:** Effects of dietary sulfur on oxidative stress markers of serum in laying hens (*n* = 8).

Item ^1^	CONT ^2^	MSM	SS	SEM ^3^	*p*-Value
GPX activity, U/L					
4 weeks	517.5	568.6	517.6	60.47	0.831
8 weeks	520.3	538.3	566.3	46.85	0.824
12 weeks	517.0	571.9	583.4	44.31	0.574
SOD activity, %					
4 weeks	74.07	95.23	95.19	7.22	0.091
8 weeks	74.88	79.57	87.86	5.45	0.282
12 weeks	85.23	103.30	92.91	5.11	0.066
TAC, mM					
4 weeks	1.30	1.54	1.52	0.13	0.423
8 weeks	1.16	1.29	1.21	0.07	0.416
12 weeks	1.57	1.59	1.60	0.08	0.961
CAT, U/mL					
4 weeks	3.06	2.59	3.19	0.36	0.541
8 weeks	2.60	2.43	2.78	0.42	0.851
12 weeks	2.45	2.96	2.63	0.23	0.341
MDA, µM					
4 weeks	23.66	17.23	17.17	3.15	0.315
8 weeks	24.62	25.40	19.70	1.60	0.083
12 weeks	30.90 ^a^	20.65 ^b^	20.85 ^b^	2.29	0.025
8-OHdG, ng/mL					
4 weeks	1.63	1.54	1.69	0.24	0.925
8 weeks	2.92	3.01	4.71	0.44	0.218
12 weeks	2.55	2.26	2.59	0.79	0.972

^a, b^ Means value without a common superscript within the same row differ (*p* < 0.05). ^1^ GPX, glutathione peroxidase; SOD, superoxide dismutase; TAC, total antioxidant capacity; CAT, catalase; MDA, malondialdehyde; 8-OHdG, 8-hydroxydeoxyguanosine. ^2^ CONT, basal diet; MSM, basal diet + methyl sulfonyl methane; SS, basal diet + sodium sulfate. ^3^ SEM, standard error of the mean.

## Data Availability

Not applicable.
